# Abrasive Wear Behavior of Cryogenically Treated Boron Steel (30MnCrB4) Used for Rotavator Blades

**DOI:** 10.3390/ma13020436

**Published:** 2020-01-16

**Authors:** Tejinder Pal Singh, Anil Kumar Singla, Jagtar Singh, Kulwant Singh, Munish Kumar Gupta, Hansong Ji, Qinghua Song, Zhanqiang Liu, Catalin I. Pruncu

**Affiliations:** 1 Mechanical Engineering Department, Sant Longowal Institute of Engineering and Technology, Punjab 148106, India; tejindergillz@gmail.com (T.P.S.); singlakiran1996@gmail.com (A.K.S.); jagtarsliet@gmail.com (J.S.); engrkulwant@yahoo.com (K.S.); 2 Key Laboratory of High Efficiency and Clean Mechanical Manufacture, Ministry of Education, School of Mechanical Engineering, Shandong University, Jinan 250061, China; munishguptanit@gmail.com (M.K.G.); hansgji@163.com (H.J.); ssinghua@sdu.edu.cn (Q.S.); melius@sdu.edu.cn (Z.L.); 3 National Demonstration Center for Experimental Mechanical Engineering Education, Shandong University, Jinan 250061, China; 4 Mechanical Engineering Department, University of Birmingham, Birmingham B15 2TT, UK; 5 Mechanical Engineering, Imperial College London, Exhibition Rd., London SW7 2AZ, UK

**Keywords:** austenitization, cryogenic, microstructure, microhardness, abrasive wear, tempering

## Abstract

Rotavator blades are prone to significant wear because of the abrasive nature of sand particles. The aim of this research work is to investigate the effect of cryogenic treatment and post tempering on abrasive wear behavior, in the presence of angular quartz sand (grain size of 212–425 μm), of rotavator blade material of boron steel (30MnCrB4). Cryogenic treatment has caused an improvement in the abrasive wear resistance and microhardness of 30MnCrB4 by 60% and 260.73%, respectively, compared to untreated material due to enhancement in hardness, the conversion of retained austenite into martensite, and the precipitation of secondary carbides in boron steel after exposure to cryogenic temperature. Economic analysis justifies the additional cost of cryogenic treatment.

## 1. Introduction

Rotavator and other earth engaging implements are needed in the farming industry, where they are used for seed bed preparation via the mixing of all crop residues to improve soil’s organic health and yield [1]. Agricultural implements wear out frequently due to abrasive action by sand/soil particles. Rotavator also gets worn out frequently due to abrasive action of sand/soil, leading to repeated replacement of its blades, which is quite uneconomical. Poor abrasive wear behavior makes farmers less efficient and plays a major role in raising the cost of agriculture [2] by adversely affecting farmers’ productivity, thereby making the whole process of agriculture economically unviable. Scientists and researchers have applied different techniques such as coatings [3,4,5,6,7,8,9,10,11,12,13,14], carburizing [15], nitriding and carbonitriding [15], boriding [15,16,17,18,19,20], and shot peening [21] to enhance the working life of rotavator blades. These techniques have shown promising results with regard to improving wear resistance in the short term, but limited data related to the long term success of these techniques is reported in the published work. These techniques largely improve a very thin layer of surface material, and chipping off this layer leads to severe wear. Therefore, there is need to explore material processing techniques for improving the abrasive wear resistance of rotavator blade material [22,23,24,25].

Conventional heat treatment (CHT) is simple, flexible, and economical, and is largely used for manipulating the microstructure, leading to alterations in the mechanical properties of the material used for earth engaging implements [26,27]. Suitable selection of the heat treatment temperature and subsequent cooling rate helps in attaining the desirable properties as per the requirements of application. Processing of materials at a low temperature in the range of −80 °C to −196 °C is another material processing technique used for achieving desirable properties in materials. Different review articles [28,29,30,31,32,33] pertaining to cryogenic processing have reported CHT’s potential in improving the properties of materials, especially ferrous-based cutting tools/implements. The efficacy of cryogenic treatment in improving the properties of nonferrous materials has also been established by different research studies [34,35,36,37,38,39]. T Singla et al. [28] have extensively examined various cryogenic cycles and their parameters for different ferrous materials. The authors have recommended using tempering post cryogenic treatment for further improving the properties of materials. The optimum number of cycles of post tempering, temperature, and time may vary for different materials. These can be optimized only based on experimental observations for different materials. Cryogenic treatment also plays a significant role in eco-friendly machining of materials, especially difficult-to-cut materials, by replacing conventional cutting fluids with liquified nitrogen [40,41,42]. The cooling medium provided to the cutting zone during cryogenic cooling evaporates immediately and comes back to the atmosphere without any pollution [40].

In this work, comparative study has been conducted amongst different thermal treatments, with a major focus on cryogenic treatment, on rotavator blade material 30MnCrB4 vis-à-vis hardness, impact strength, and abrasive wear resistance. The alterations in mechanical behavior have been correlated with microstructural evolution. Economic analysis justifying the additional cost of cryogenic treatment due to its improvement in the performance of rotavator blade material has been reported.

## 2. Materials and Methods

### 2.1. Untreated Material

In the present work, low-carbon boron steel (DIN 30MnCrB4) is selected as an untreated material, which is widely used for manufacturing of rotavator blades [43,44]. Boron steel exhibits better hardness, sliding, and abrasive wear resistance due to the presence of hardenability enhancers like chromium and boron, as compared to high-carbon steel [25]. The chemical composition of as-received material is tested using optical emission spectrometer (GNR Solaris CCD-Plus, Torino, Italy). The obtained chemical composition along with standard composition are presented in Table 1.

### 2.2. Methods

**Figure 1:**
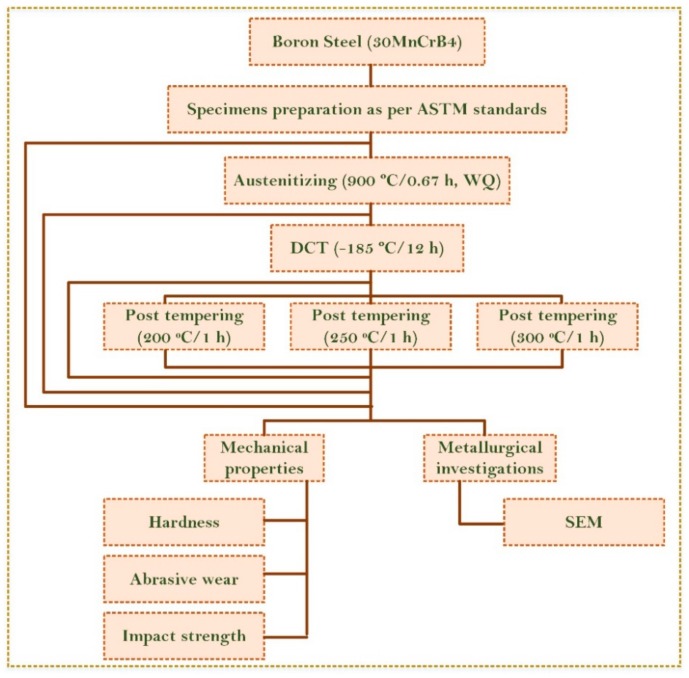
Schematic plan of thermal treatments and different investigations.

The schematic plan depicting the various thermal treatments and mechanical and metallurgical investigations is given in Figure 1.

#### 2.2.1. Austenitization

A pilot study [45,46] was undertaken to decide about the parameters of austenitization, such as austenitic temperature, soaking period, and quenching medium. L9 Taguchi DOE (design of experiments) was adopted for this purpose. The design factors along with three levels for each factor are shown in Table 2.

The output parameters were abrasive wear volume loss, hardness, and impact toughness. It was observed that optimum results were attained corresponding to the austenitization temperature of 900 °C with a soaking period of 40 min and water as a quenching media. The complete details of the pilot study are given in research study by Singh et al. [46]. Accordingly, in this work, these optimized austenitization parameters have been chosen for further investigation of low-temperature treatment. Muffle furnace (Jupiter engineering works, New Delhi, India) has been used for austenitization, and all specimens have been properly cleaned ultrasonically using acetone before heat treatment to remove the oil, dust, foreign particles, and grease from the surface of specimens. The muffle furnace was allowed to reach 900 °C, and specimens were placed inside the muffle furnace. The specimens were held at that temperature for 40 min and then water-quenched.

#### 2.2.2. Deep Cryogenic Treatment

Austenitization was immediately, without any time lag, followed by deep cryogenic treatment (DCT), and its parameters are reported in Table 3. All the specimens were again ultrasonically cleaned with acetone before deep cryogenic processing. DCT was conducted using cryogenic processor (3241-1-SAMSON, Super Cryogenic Systems Pvt. Ltd., Noida, India). Cryogenic treatment set up was brought to the desired temperature using computer controls in a well-insulated chamber with liquid nitrogen as a working medium.

#### 2.2.3. Post Tempering

**Figure 2:**
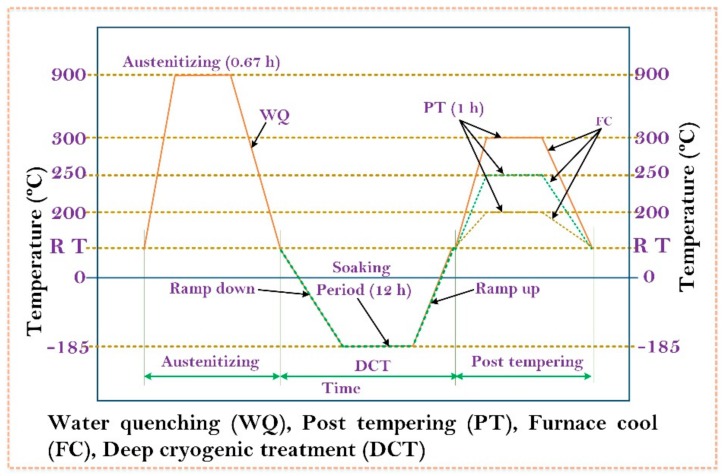
Thermal treatment cycles.

Post tempering treatment was given to all the cryogenically treated specimens in three different groups at three tempering temperatures. The three sets of specimens were subjected to post tempering cycle at 200 °C, 250 °C, and 300 °C, respectively, for 1 h. The complete details of austenitization, DCT, and tempering cycles are presented in Figure 2. The various thermal cycles adopted for the present work and coding of specimens are detailed in Table 4.

### 2.3. Metallurgical Investigation

Scanning electron microscopy, using JSM 6510LV (JEOL, Tokyo, Japan) was conducted for visualization of microstructure and study of fractography. For microstructure, the specimens were etched first with freshly prepared etchant 4% picric (picric acid 4 gm and ethanol 96 mL) by swabbing for 20–30 s followed by distilled water rinsing. They were then etched with 2% nital (nitric acid 2 mL and ethanol 98 mL) by swabbing for 15–25 s followed by distilled water rinsing. The specimens were dried in air and observed for obtaining SEM images.

### 2.4. Mechanical Properties

The micro hardness measurement was conducted as per ASTM E384 using digital micro Vickers hardness tester (RMHT-201, Radical, Ambala Cantt, India). Measurements were taken at five different locations of UT, CHT, CDCT-T0, CDCT-T1, CDCT-T2, and CDCT-T3 specimens. Four readings were taken at the periphery and one at the center of specimen. Vickers micro-hardness measurement was conducted using a 4.9 N load for a total cycle time of 40 s with a dwell time of 20 s. The average of five observations was computed and reported for this work. 

Charpy V-notched impact test was performed as per ASTM E23-07a [47] using sub-size specimens (7.5 × 10 × 55) mm. Three specimens for each treatment CHT, CDCT-T0, CDCT-T1, CDCT-T2, and CDCT-T3 were tested, and observations with average value are reported in this work.

**Figure 3:**
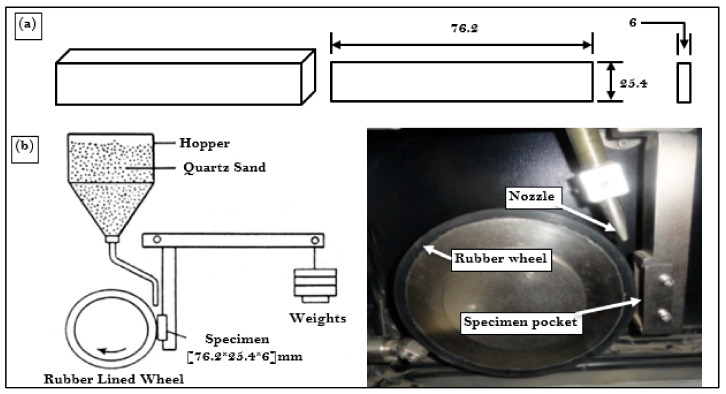
Schematic view of (**a**) abrasive wear test specimen and (**b**) dry sand abrasion test rig.

Specimens of size (76.2 × 25.4 × 6) mm, as shown in Figure 3a, were prepared using tabletop cutting equipment (Labotom 5, Struers, Ballerup, Denmark) for abrasive wear testing. Abrasive wear test was performed as per ASTM G65 [48] on the specimens of UT, CHT, CDCT-T0, CDCT-T1, CDCT-T2, and CDCT-T3 using abrasive wear test rig (TR-50-M7, Ducom, Bangalore, India) shown in Figure 3b. The parameters selected for abrasive wear testing are shown in Table 5. The values of speed, rotational speed of wheel, and sliding distance were selected to emulate the actual agricultural field conditions. Specimens were ultrasonically cleaned before the test to remove any foreign particles from the specimens. The weight of each specimen, before and after the experiment, was determined by using precision weighing balance (Citizen CY204, Denver Instruments GmbH, Denver, Germany) with a least count of 0.0001 g. The resulting weight loss was converted into abrasive wear loss in mm^3^. Each experiment was performed three times to minimize the experimental errors.

**Figure 4:**
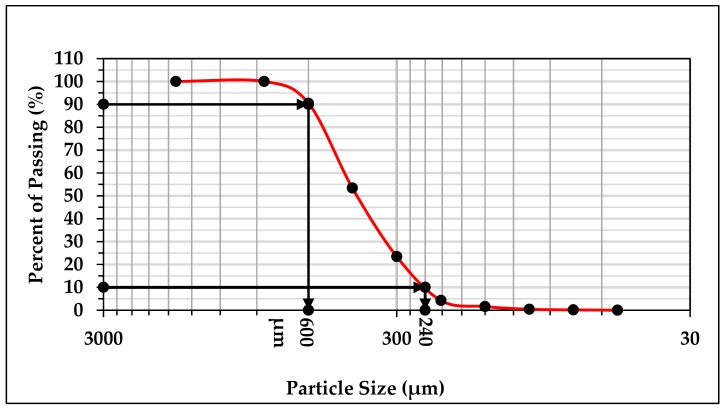
Particle size distribution curve.

The abrasive wear study of the material was influenced by the size and morphology of abrasive sand. As per Avery [49], abrasive wear behavior of material was significantly affected by the sand particle size. The quartz sand was arranged for the test and standard sieve shaker (Kelsons Engineers and Fabricators, Kohlapur, India) was used to find out the particle size distribution. Particle size distribution for the used sand is presented in Figure 4. d10 and d90 of the quartz sand used for wear test were computed and marked on the particle distribution curve shown in Figure 4. It is clear from particle distribution curve that 80% of the quartz sand particles lie between 240 μm and 600 μm with an average particle size of 430 μm. The size of sand available in fields also lie in the same range as reported by Singh et al. [50].

**Figure 5:**
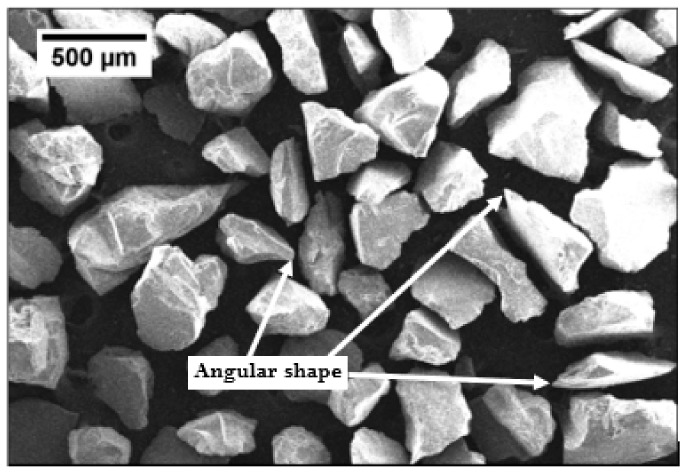
The geometry of quartz sand.

To obtain morphology of the quartz sand particle, scanning electron microscopy (JSM 6510LV, JEOL, Tokyo, Japan) was used for the present study, and same is shown in Figure 5. The hardness value of quartz sand is determined based on past studies [50,51] and found to be in the range of 1070–1200 HV. It is clearly shown in the SEM image of quartz sand that particles are highly angular, which is responsible for aggressive wear [52,53].

## 3. Results and Discussions

### 3.1. Microstructure

**Figure 6:**
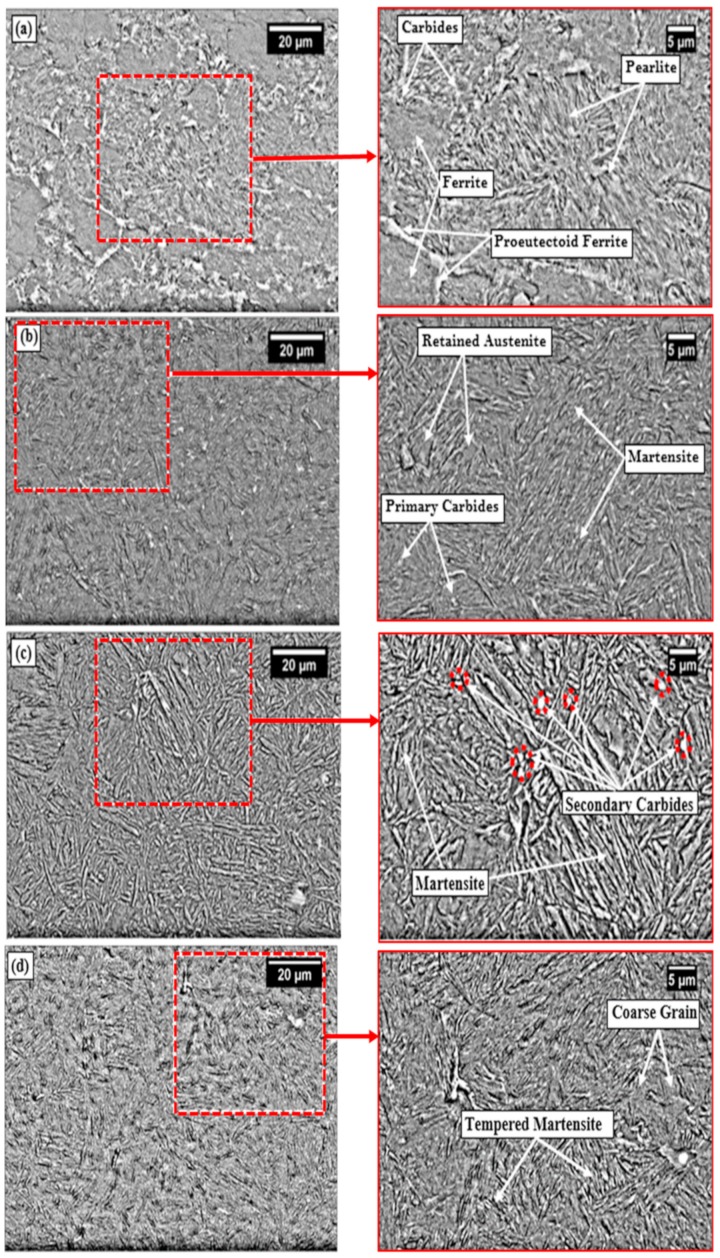
SEM microstructures of (**a**) untreated, (**b**) conventional heat-treated (900 °C/0.67 h, water-quenched), (**c**) conventional heat-treated (900 °C/0.67 h, water-quenched) + deep cryogenic treatment (−185 °C/12 h), and (**d**) conventional heat-treated (900 °C/0.67 h, water-quenched) + deep cryogenic treatment (−185 °C/12 h) + tempering (200 °C/1 h, furnace-cooled).

The SEM micrograph of UT sample is characterized by presence of ferrite and pearlite structure, as shown in Figure 6a. The network of ferrite appeared as flat surface and pearlite as needle pattern surface [45]. During austenitization, ferrite and pearlite phases were transformed into austenite phase. Austenite is quite soft as compared to pearlite, and started transforming into martensite upon quenching in water. Formation of martensite has led to enhancement of hardness [54,55]. A sufficient fraction of retained austenite (RA) was also present upon quenching to room temperature, which is quite evident from microstructure of austenitized boron steel shown in Figure 6b. Few primary carbides and martensite formation have been identified and marked on SEM image shown in Figure 6b. During deep cryogenic treatment, retained austenite left after CHT was transformed into martensite. A significant fraction of martensite phase is lucidly visible in the SEM image shown in Figure 6c. Formation of secondary carbides on exposure to soaking temperature was also visible in microstructure of CDCT-T0 specimen. Soaking for long time helped in bringing more uniformity in distribution of secondary carbides, as shown in white spot encircled with red color, which led to improvement in hardness and wear resistance of the material [29,56,57,58]. The presence of martensite and secondary carbides in CDCT-T0 specimen were identified and marked in its SEM micrograph, as shown in Figure 6c. SEM micrograph of tempered post cryogenic treatment boron steel is shown in Figure 6d. It is evident from SEM image of CDCT-T1 specimen that post tempering resulted into grain coarsening and martensite decomposition. Transformation of martensite into tempered martensite and mechanical mixture of phases resulted in reduction of hardness and wear resistance of the CDCT-T1 specimen in comparison with that of CDCT-T0 sample. The results are in agreement with those reported by Kalsi et al. [32].

### 3.2. Hardness

**Figure 7:**
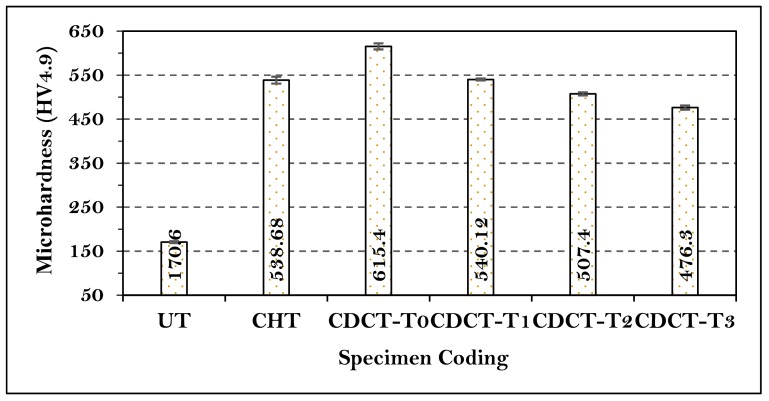
Microhardness of untreated and treated material.

The Vickers micro-hardness of UT, CHT, CDCT-T0, CDCT-T1, CDCT-T2, and CDCT-T3 specimens was measured at five different locations for each sample, and the mean value was computed and shown in the form of bar chart in Figure 7. From the results drawn in Figure 7, it is clear that hardness increased in the specimens CHT (215.76%), CDCT-T0 (260.73%), CDCT-T1 (216.60%), CDCT-T2 (197.42%), and CDCT-T3 (179.19%), as compared to UT sample. The maximum improvement in the hardness was observed in CDCT-T0 sample due to conversion of RA into martensite and formation of secondary carbides after deep cryogenic treatment [57,59,60,61].

### 3.3. Impact Strength

**Figure 8:**
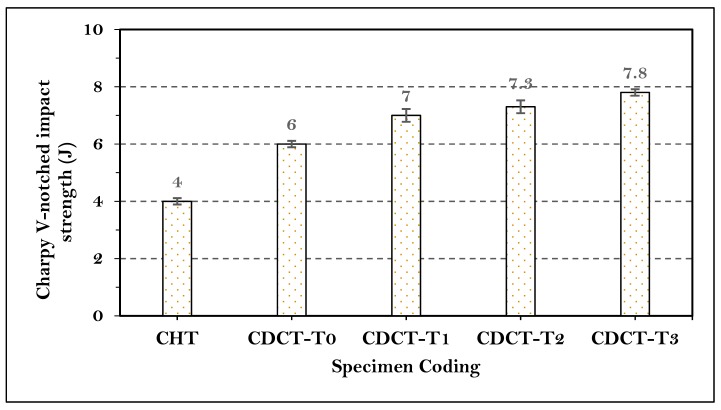
Charpy V-notch impact strength results of treated material.

A set of three specimens were prepared for analyzing the impact strength of material, and the mean of these three values was computed. The average value has been drawn in the form of bar chart as shown in Figure 8. From the results shown Figure 8, it is clear that impact strength increased in the specimens CDCT-T0 (50%), CDCT-T1 (75%), CDCT-T2 (82.5%), and CDCT-T3 (95%), compared to CHT sample. The maximum increase in the impact strength was observed in CDCT-T3 sample due to transformation of martensite, which formed during hardening, into tempered martensite along with secondary carbide formation and removal of residual stresses due to tempering post cryogenic treatment. The nucleation of carbides and precipitation of finer carbides increase the impact strength of material after deep cryogenic treatment. During cryogenic treatment, fine platelets of martensite are formed from the retained austenite, and these platelets promote the precipitation of fine carbides by a diffusion mechanism during tempering [58].

**Figure 9:**
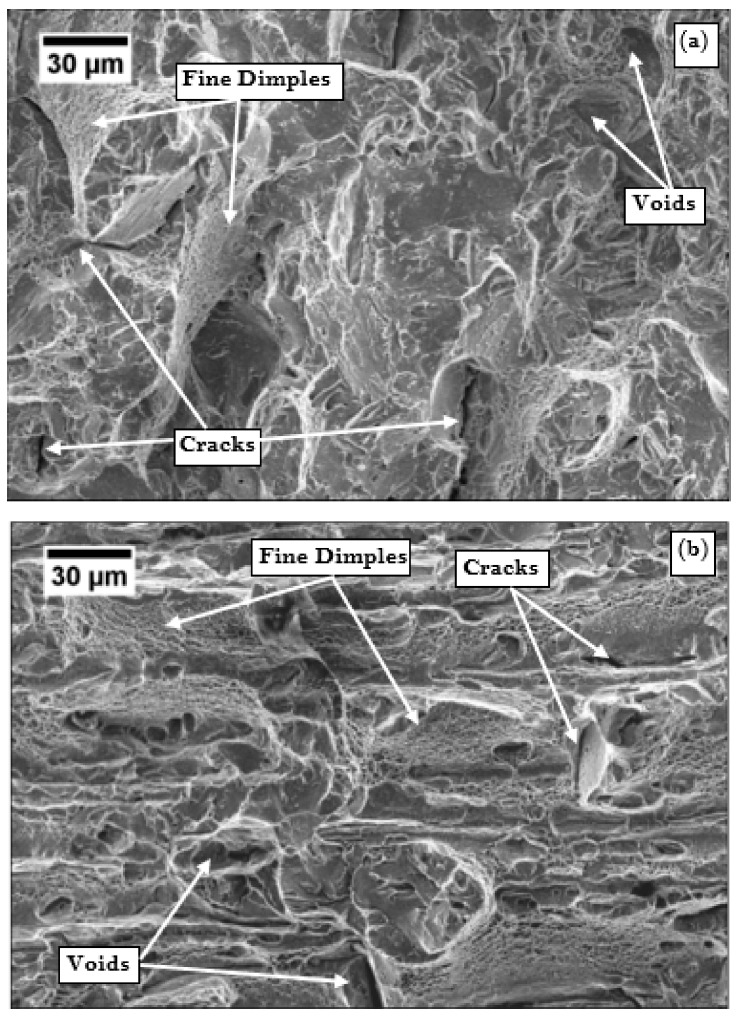

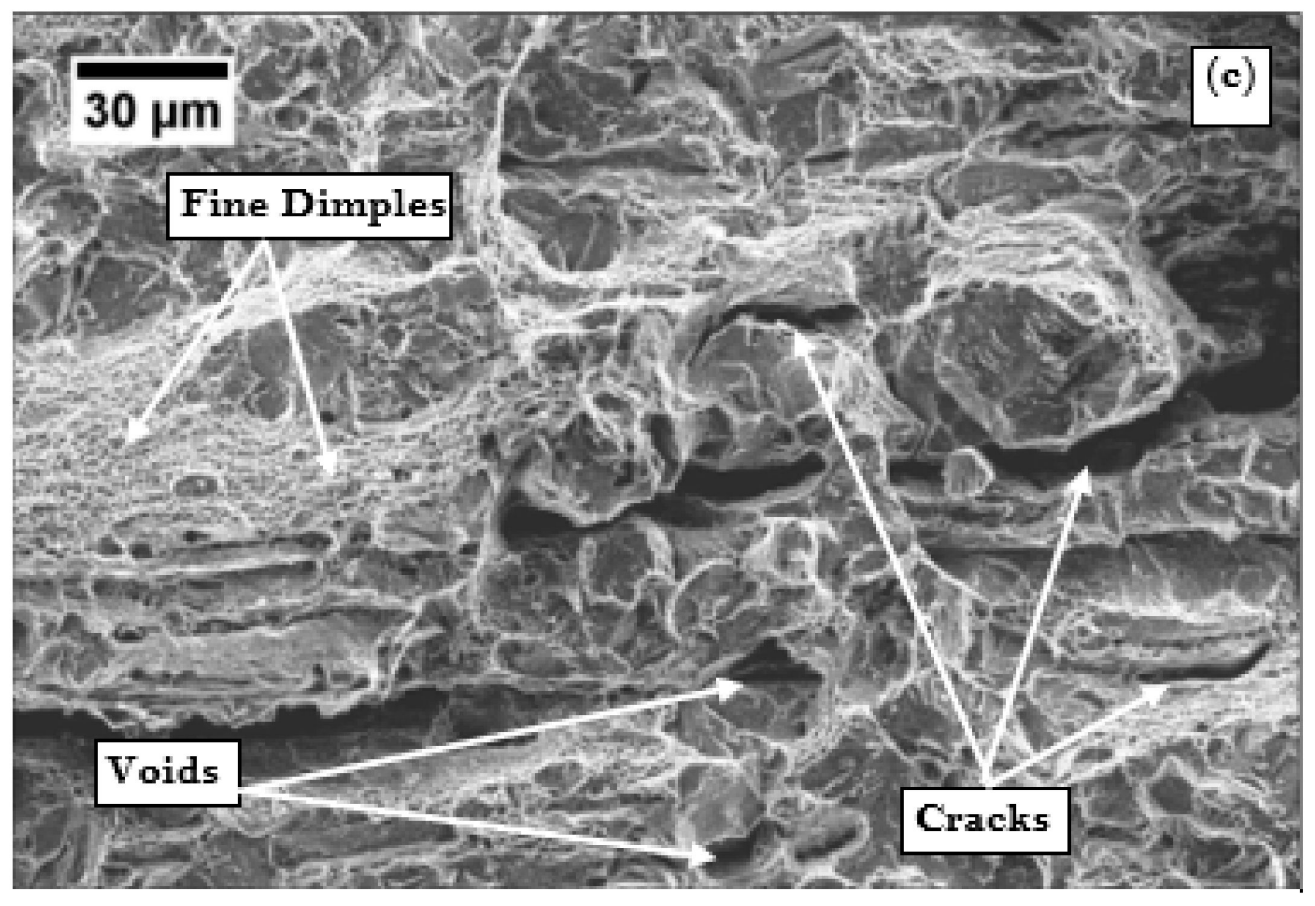
SEM fractography images of impact samples of boron steel: (**a**) conventional heat-treated (900 °C/0.67 h, water-quenched), (**b**) conventional heat-treated (900 °C/0.67 h, water-quenched) + deep cryogenic treatment (−185 °C/12 h), and (**c**) conventional heat-treated (900 °C/0.67 h, water-quenched) + deep cryogenic treatment (−185 °C/12 h) + tempering (300 °C/1 h, furnace-cooled).

SEM fractography of impact specimens was conducted to examine the nature of fractured surface. Impact strength of CHT samples was the lowest, and cryogenic treatment caused improvement in impact strength by 50%, which was further enhanced by tempering. The SEM fractography images of the CHT, CDCT-T0, and CDCT-T3 specimens taken at the central region of the Charpy V-notch impact test specimens are shown in Figure 9. Flat regions surrounded by the fibrous regions, which were formed by tearing ridges in the CHT specimen shown in Figure 9a. The presence of rippled morphology with very few voids and few visible cracks accounted for its low impact energy absorption capacity. In the CDCT-T0 specimen shown in Figure 9b, cracking was observed with severe bubble/dimples coalescence along the grain boundaries. The presence of micro dimples on the fracture facets shows that a considerable amount of plastic deformation occurred prior to the fracture, resulting in absorption of increased impact energy of the cryo-treated specimen. In the CDCT-T3 specimen shown in Figure 9c, more cracking was observed is evident, along with dense micro dimples coalescence formation. The presence of many microcracks and crack branching with dense micro dimples indicates more plastic deformation occurred before fracture, resulting in increase in the impact strength of post-tempered cryogenic-treated specimens [58,62].

### 3.4. Abrasive Wear

**Figure 10:**
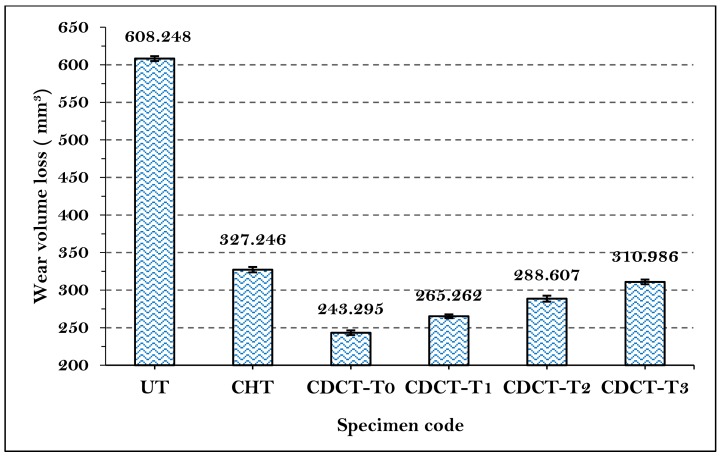
Comparison of abrasive wear loss of untreated and treated material.

Abrasive wear volume loss was computed from the weight loss and density of material (7.85 g/cm^3^) for each sample. The average abrasive wear volume loss was plotted in the form of bar chart as shown in Figure 10. It is clear from the results shown in Figure 10 that abrasive wear resistance increased in the specimens CHT (46.20%), CDCT-T0 (60.00%), CDCT-T1 (56.39%), CDCT-T2 (52.55%), and CDCT-T3 (48.87%), compared to UT sample. The maximum improvement in abrasive wear resistance was observed in CDCT-T0 sample due to conversion of RA into martensite and formation of secondary carbides after deep cryogenic treatment [57,61]. Post tempering increased wear volume loss with increase in tempering temperature attributable to decrease in hardness [63].

#### Worn Surface Morphology

**Figure 11:**
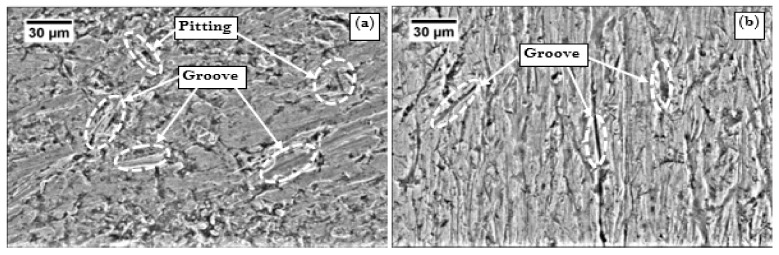
Abraded surfaces of: (**a**) untreated 30MnCrB4 Steel {UT} and (**b**) conventional heat-treated (900 °C/0.67 h, water-quenched) + deep cryogenic-treated (−185 °C/12 h) 30MnCrB4 steel {CDCT-T0}.

Since the abrasive wear volume loss was maximum in UT samples and minimum in CDCT-T0 boron steel, SEM images of worn surface of UT and CDCT-T0 specimens of boron steel are reported in Figure 11. The SEM image of UT specimen is marked by the pitting, deep, and broad grooves resulting from rolling and sliding motion of abrasive sand particles between the rubber wheel and the specimen. The sharped edge angular shaped abrasive particles pushed the material to both sides of abrasive groove, due to the repeated action of ploughing. Some material of specimen that drops out due to exhaustion of plasticity was responsible for pitting. These sharp-edge abrasive sand particles penetrated deep into the material of specimen, resulting in a large amount of material removal from specimens by changing the mode from rolling to sliding by abrasive sand particles. In steel with lower hardness, rolling mechanism is dominant while in steel having higher hardness, grooving is dominant feature on worn surfaces [51]. The continuous rubbing of abrasive sand against specimen surface also resulted in the formation of grooves. The results are in agreement with those reported by Chand et al. [64].

During sliding distance of 1900 m, some particles of specimen’s material dropped out, generating pitting along with wide and deep grooves on the surface of the specimen due to their plastic deformation with the continuous and repeated ploughing action, which was responsible for large wear loss of the material [56,65]. It is clearly visible in the SEM image of UT specimen presented in Figure 11a. In SEM image of CDCT-T0 specimen, presented in Figure 11b, few shallow groove formations on the abraded surface of the specimen were observed. Number of grooves as well as depth of grooves was smaller than that in UT specimen, leading to significant improvement in the morphology of worn surface. Improvement in the hardness of the material reduced the ploughing depth, and offered stronger support for carbides to inhibit its spalling and could prevent large grooves forming during abrasive action of quartz sand. Improvement in abrasive wear behavior was due to increase in hardness, RA conversion into martensite, formation of secondary carbides, and their more uniform distribution, which ultimately led to reduction in abrasive wear volume loss [56,61,63,66,67].

### 3.5. Economic Analysis

Economic analysis has been done to evaluate the expected improvement in performance of rotavator blade during field operation against the additional cost quantified in Indian Rupees (Rs.) due to cryogenic treatment. The details are as follows:
The cost of one blade (including cost of hardening)=Rs. 215Size of rotavator=07 FeetTotal number of blades =48Weight of one blade=1.04 KgNumber of blades to be cryogenically treated in one lot =5 × 48 = 240Additional cost for proposed cryogenic treatment on one lot =Rs. 7800Additional cost/blade=7800/240 = Rs. 32.50Percentage improvement in wear resistance of blade after cryogenic treatment in comparison with CHT (hardened)=25.7%Percentage increase in cost per blade due to cryogenic treatment=15.12%

It is evident from the economic analysis that improvement quantified in monetary terms is more that the additional cost due to cryogenic treatment. Therefore, deep cryogenic treatment processing of rotavator blades made of boron steel is recommended for improving the abrasive wear resistance and other mechanical properties.

## 4. Conclusions

The effect of cryogenic treatment and post-tempering on rotavator blade material were analyzed. The following conclusions were drawn:

(1) During deep cryogenic treatment, retained austenite left after conventional heat treatment was transformed into martensite. Cryogenic treatment also resulted in formation of secondary carbides and helped in bringing more uniformity in distribution of secondary carbides. Tempering post cryogenic treatment led to grain coarsening and martensite decomposition;
(2) Hardness of cryotreated (CDCT-T0) specimen was improved by 260.73% compared to UT material, due to the formation of martensite along with the precipitation of secondary carbides. Tempering post cryogenic treatment caused reduction in hardness due to grain coarsening and martensite decomposition;
(3) Impact strength of the cryotreated (CDCT-T0) specimen was augmented by 50% in comparison with CHT specimen, due to increasing the nucleation of carbides, which facilitated the precipitation of a higher number of fine carbides during cryogenic treatment, resulting in a higher impact strength of material. Post tempering enhanced the impact strength, which further increased with higher tempering temperature;
(4) Abrasive wear volume loss in cryotreated (CDCT-T0) specimens were reduced by 60% compared to UT samples, owing to improvement in hardness, RA conversion into martensite, and the formation of secondary carbides. Tempering post cryogenic treatment resulted in decline in abrasive wear resistance;
(5) The additional cost of 15.12% was incurred due to cryogenic treatment, whereas the expected augmentation in wear resistance of rotavator blade material was 25.70%. The economic analysis clearly justified the additional cost of cryogenic treatment.

## Figures and Tables

**Table 1:** Chemical composition of boron steel (30MnCrB4) in weight (%).

Elements	As Received	As Per Standards
C	0.29	0.24–0.30
Mn	1.23	1.10–1.40
Si	0.221	0.40
P	0.032	0.030
Cr	0.306	0.30–0.60
B	0.002	0.0008–0.0050
Fe	balance	balance

**Table 2:** Factor and level for DOE (design of experiments) of austenitization.

Factors	Levels		
	1	2	3
A: Medium	Water	Oil	Oil-Water
B: Temperature (°C)	800	850	900
C: Time (min)	20	30	40

**Table 3:** Description of deep cryogenic treatment (DCT) process parameters.

Sr. No.	Parameters	Level
1	Soaking temperature (°C)	−185
2	Cooling rate (°C/min)	0.5
3	Soaking period (h)	12
4	Heating rate (°C/min)	0.5

**Table 4:** Thermal treatment schedules and coding of specimens.

Sr. No.	Type of Treatment	Specimen Coding
1	Untreated Material	UT
2	CHT (900 °C/0.67 h, WQ)	CHT
3	CHT (900 °C/0.67 h, WQ) + DCT (−185 °C/12 h)	CDCT-T0
4	CHT (900 °C/0.67 h, WQ) + DCT (−185 °C/12 h) + Tempering (200 °C/1 h, FC)	CDCT-T1
5	CHT (900 °C/0.67 h, WQ) + DCT (−185 °C/12 h) + Tempering (250 °C/1 h, FC)	CDCT-T2
6	CHT (900 °C/0.67 h, WQ) + DCT (−185 °C/12 h) + Tempering (300 °C/1 h, FC)	CDCT-T3

Note: WQ—water quenching, FC—furnace cooling, CHT—conventional heat treatment, UT—untreated.

**Table 5:** Parameters used for three-body abrasion wear test (DSRW).

Parameters	Unit	Value
Load	N	130
Speed	m/s	3.17
Rotational speed of wheel	rpm	200
Abrasive flow rate	g/min	300
Abrasive size	µm	212–425
Sliding distance	m	1900
